# Supplementary figures and images for: Exploring the druggable space around the Fanconi anemia pathway using machine learning and mechanistic models
Source: BMC Bioinformatics. 2019 Jul 2;20:370. doi: 10.1186/s12859-019-2969-0 (PMC6604281; doi:10.1186/s12859-019-2969-0)

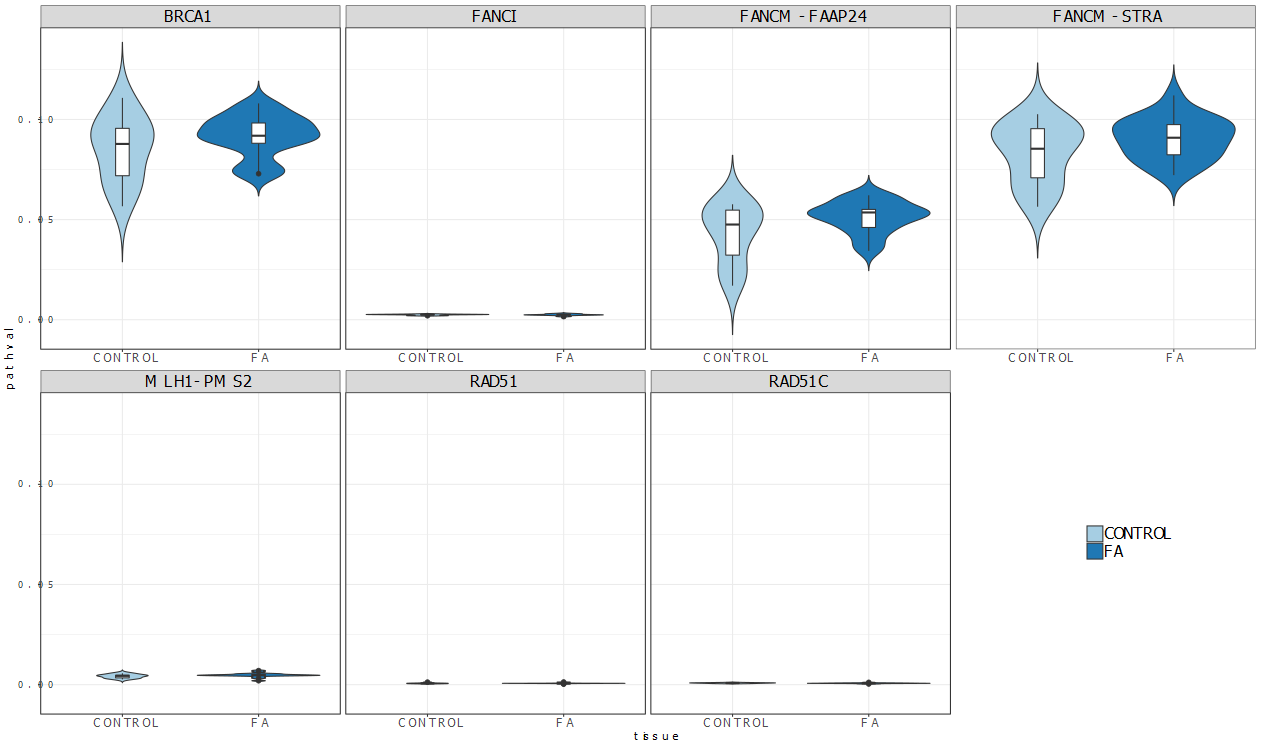

Supplement: Supplementary file 3 — Figure S3. Distribution of circuit activities in the FA KEGG pathway. Distribution of activities in the seven circuits of the FA KEGG pathway observed in the comparison between healthy and FA bone marrow cells. (TIF 218 kb) [file 12859_2019_2969_MOESM3_ESM.tif]
